# Supplementary material for: Evaluation of deep learning-based multiparametric MRI oropharyngeal primary tumor auto-segmentation and investigation of input channel effects: Results from a prospective imaging registry
Source: Clin Transl Radiat Oncol. 2021 Oct 16;32:6–14. doi: 10.1016/j.ctro.2021.10.003 (PMC8570930; doi:10.1016/j.ctro.2021.10.003)
Supplement: Supplementary data 1 [file mmc1.docx]

**Appendices**

**Appendix A. Supplementary Methods**

*1. DCE Parametric Map Generation*

Motion correction and noise suppression were applied using simultaneous spatial and temporal higher-order total variations [1]. The arterial input function of the contrast agent entering the tissue was determined individually, and a T1 map was calculated to convert signal intensity into a concentration-time course for use in the Tofts model. We utilized a biexponential and bilinear function for arterial input function fitting. Ktrans and Ve estimation was performed on a pixel-by-pixel basis using a linearization equation of the Tofts model. A linear least-squares method was then used to acquire the final parameters.

*2. Deep Learning*

Throughout our analysis, we adhered to the standardized artificial intelligence in medicine reporting guideline CLAIM [2] to ensure the reproducibility and rigor of our methodologic approach. The goal of this study was to create an oropharyngeal cancer (OPC) primary gross tumor volume (GTVp) auto-segmentation tool using multiparametric MRI (mpMRI) and determine the impact of individual mpMRI channels on model performance.

DICOM MRI images and DICOM RT GTVp structures were converted to Neuroimaging Informatics Technology Initiative (nifti) format for use in deep learning models using the DICOMRTTool v. 0.3.13 Python package [3].

*Model Architecture:* A DL convolutional neural network based on the 3D residual U-net architecture [4,5] was implemented in the Medical Open Network for Artificial Intelligence (MONAI) software package [6]. The images acted as variable-channel inputs to the models. The network consisted of four convolution blocks in the encoding and decoding branches and a bottleneck convolution block between the two branches. All convolution layers used a kernel size of 3 except for one convolution layer in the bottleneck, which used a kernel size of 1. The number of output channels for each convolution layer is shown in **Figure 1C** of the main text above each layer. Each convolution block in the encoding branch was composed of a two-strided convolution layer and a residual connection that contained a two-strided convolution layer and a one-strided convolution layer. In the bottleneck, the residual connection contained two one-strided convolution layers. In the decoding branch, each block contained a two-strided convolution transpose layer, a one-strided convolution layer, and a residual connection. Batch normalization and parametric rectified linear unit activation functions were used throughout the architecture. Parametric rectified linear unit activation functions were chosen since they have been found to improve upon the rectified linear unit activation function, with increased performance for image-related tasks [7]. A softmax function was applied to the two-channel output to generate the GTVp segmentation mask (0 = background, 1 = tumor).

*Model Implementation:* Processed images were cropped to four random fixed-sized patches of size (96, 96, 96) per patch, with the center being considered foreground (i.e., GTVp, positive) or background (i.e., not GTVp, negative), with a 50% probability for both positive and negative cases. We implemented a batch size of two patients, resulting in a total of eight patches of images. The GTVp mask was used as the ground truth target to train the segmentation model. We implemented data augmentation on both the image and mask patches to mitigate overfitting, which included random horizontal flips of 50% and random affine transformations with an axial rotation range of 12 degrees and a scale range of 10%. Image processing and data augmentation were performed by the image transformation packages provided by the MONAI framework [6]. We implemented an Adam optimizer with a Sørensen-Dice similarity coefficient (DSC) loss function. The model was trained for 700 iterations with a learning rate of 2 x 10^-4^ for the first 550 iterations and 1 x 10^-4^ for the remaining 150 iterations.

**Appendix B. Additional Visual Analysis of Select Auto-Segmentation Results**

In this appendix, we will explore potential causes of major discrepancies between deep learning generated segmentations and ground truth segmentations for select oropharyngeal cancer (OPC) patients that are of interest in our analysis. Specifically, we explore an HPV-negative case (Case 1) and two failure cases (Case 2 and Case 3). For these analyses, we focus on results from the T2-weighted (T2) baseline model, the overall best model in our analysis, i.e., the T2-weighted+T1-weighted (T2+T1) model, and the overall worst model in our analysis, i.e., the five input channel (ALL) model.

*Case 1: HPV-Negative Tumor*

In this case, we further investigate the only patient with a histologically confirmed HPV-negative tumor used in our analysis. The patient was a 70-year-old Caucasian male with a bilateral (left side dominant) base of tongue HPV-negative tumor, stage IVA (T2, N2b, M1), treated with concurrent chemoradiotherapy (cisplatin-based chemo with proton radiotherapy). For the T2 baseline model, compared to the mean DSC performance (across all patients) the performance for the non-HPV-associated tumor case is notably lower (0.56 vs. 0.72). This trend is also apparent for the ALL model, with lower performance for the non-HPV associated tumor case (0.53 vs. 0.71). Conversely, for the T2+T1 model, the mean DSC performance compared to the non-HPV-associated tumor case is similar (0.69 vs. 0.73).

It can be observed that all three investigated models had difficulties with more inferior slices near the lower level of the epiglottic vallecula (**Fig. B1, B2, B3**, 1st row). This could be expected as most cases used for model training did not have tumors that extended inferiorly to this level for ground truth segmentations. Moreover, the T2 baseline model had difficulty with more superior slices, overestimating the tumor edges, while the T2+T1 and ALL models were more conservative, leading to a more accurate volumetric overlap (**Fig. B1, B2, B3**, 3rd row). Finally, and perhaps most interestingly, the T2 baseline model erroneously segmented areas of the right palatine tonsil, which were exacerbated in the ALL model where additional palatine tonsil and uvula areas were segmented (**Fig. B1, B3**, 4th row). It is possible the additional signal in functional parametric maps in these areas contributed to the worsening of spurious voxel predictions. The incorrect palatine tonsil segmentations were not present in the T2+T1 model (**Fig. B2**, 4th row). Overall, these results may indicate the T2+T1 model is better suited for HPV-negative tumors, but also may be a byproduct of overall improved performance for OPC tumors generally.

HPV-negative tumors tend to, on average, display more ulceration and necrosis, have less well-defined borders, and be less exophytic compared to HPV-positive tumors [8]. These distinct morphologic features are reflected in this case and may indicate situations where the models had difficulties in comparison to the majority of data that was used in model training. However, it is difficult to draw definitive conclusions on why performance would be different for HPV-negative cases compared to the HPV-positive cases in this study based on this one example. Therefore, future studies should investigate the difference in segmentation performance between HPV-positive and -negative cases when evaluating OPC tumor DL auto-segmentation systems.

**
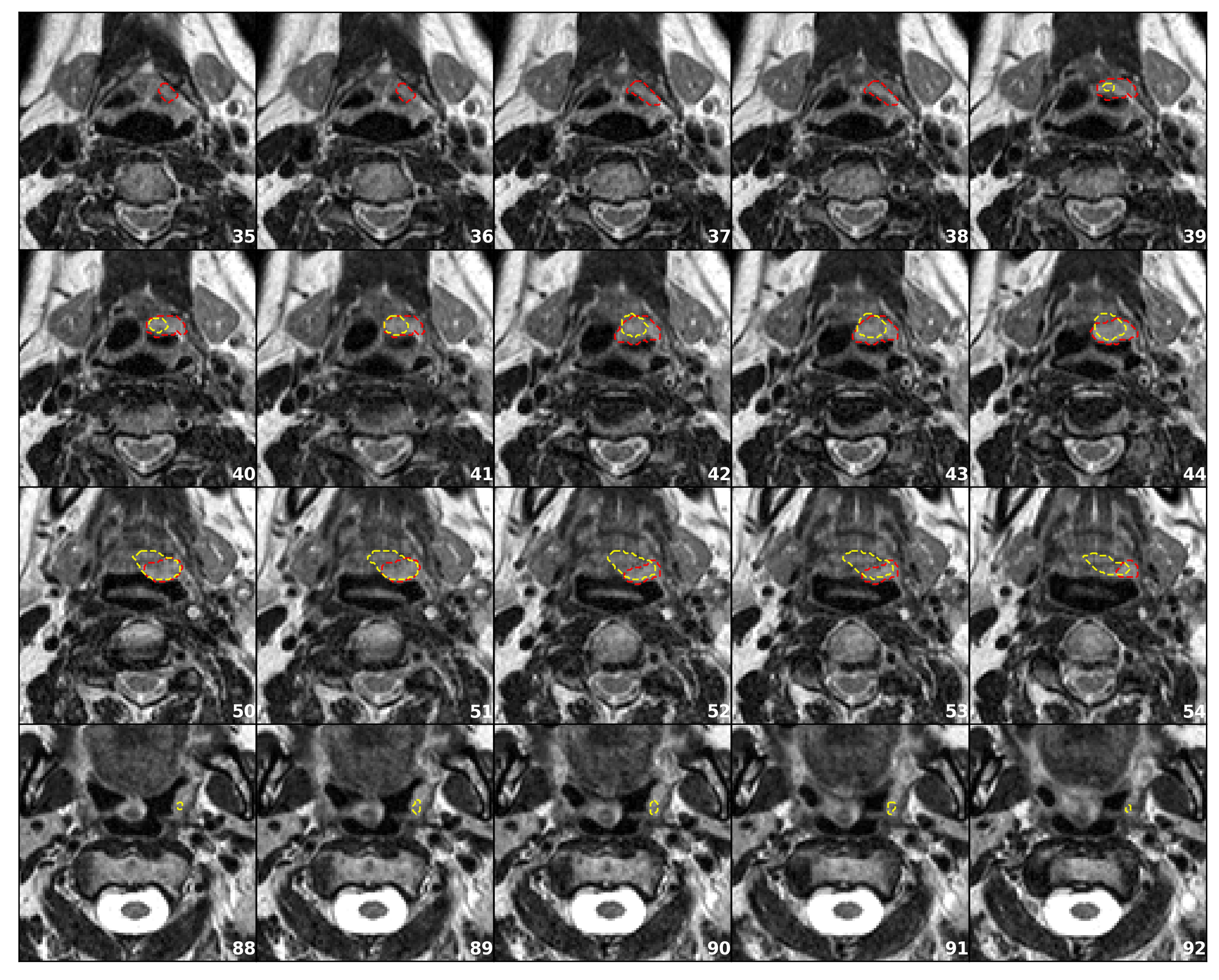
**

**Figure B1.** Full view 2D axial slice representations of ground truth segmentations (red dotted outline) and predicted segmentations from T2-weighted model (yellow dotted outline) for HPV-negative tumor. The slice locations of the segmentations are shown in the bottom right corners of each image. The DSC for this model was 0.56 for this case.


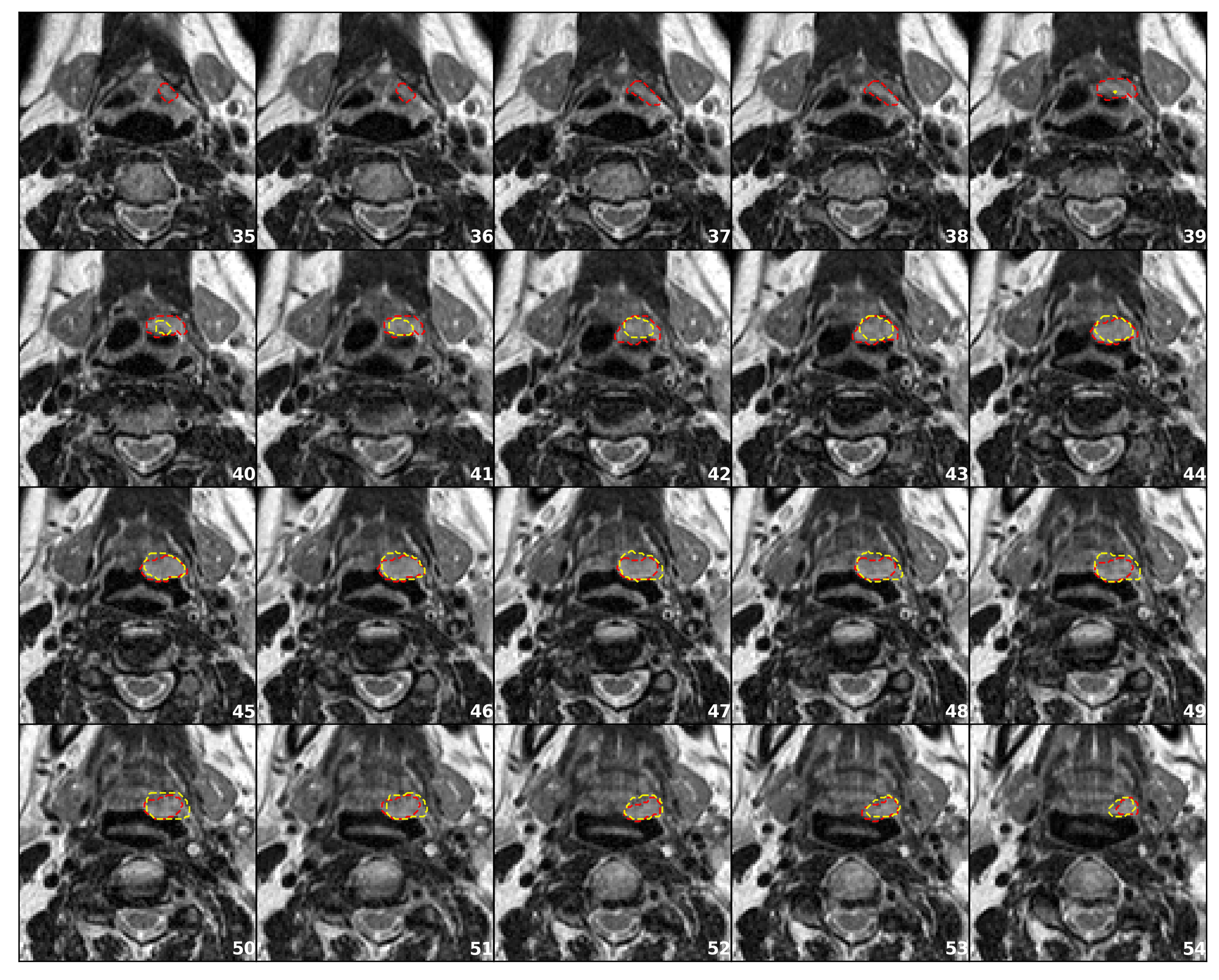


**Figure B2.** Full view 2D axial slice representations of ground truth segmentations (red dotted outline) and predicted segmentations from T2-weighted+T1-weighted model (yellow dotted outline) for HPV-negative tumor. The slice locations of the segmentations are shown in the bottom right corners of each image. The DSC for this model was 0.69 for this case.


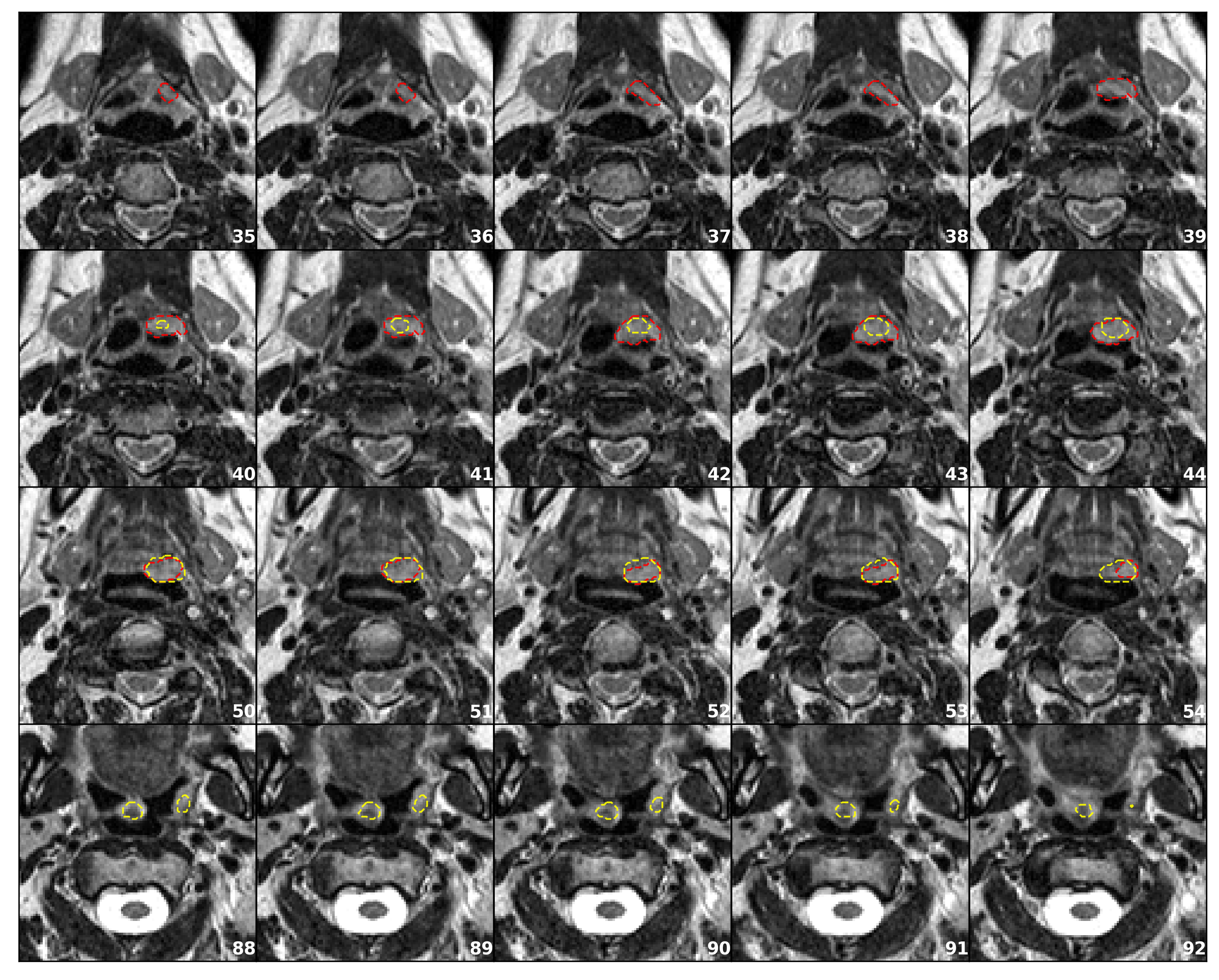


**Figure B3.** Full view 2D axial slice representations of ground truth segmentations (red dotted outline) and predicted segmentations from all 5 channels model (yellow dotted outline) for HPV-negative tumor. The slice locations of the segmentations are shown in the bottom right corners of each image. The DSC for this model was 0.53 for this case.

Notably, the clinical evaluation and Turing test results for the T2+T1 model for this case were not overtly worse than ground truth (**Table B1**), as the only observer to rate the deep learning generated segmentation lower than the ground truth segmentation was Observer 1, which was consistent with their evaluations for the entire dataset (see main manuscript). Therefore, on clinical observation, this model is qualitatively consistent in performance for the evaluated HPV-negative case compared to the mean results for all the patients in our cohort.

**Table B1.** Clinical evaluation and Turing test results for three physician expert observers for HPV-negative case. Each observer was asked to score blinded ground truth (GT) or deep learning (DL)-generated segmentations on a 4-point Likert scale (1 = requires corrections, large errors; 2 = requires corrections, minor errors; 3 = clinically acceptable, errors not clinically significant; 4 = clinically acceptable, highly accurate) and asked to identify the source of the segmentation (GT or DL). DL-generated segmentations corresponded to the best DL model tested (T2-weighted + T1-weighted).

| **Observer** | **GT Score** | **GT Source** | **DL Score** | **DL Source** |
| --- | --- | --- | --- | --- |
| 1 (Radiologist) | 3 | DL | 2 | DL |
| 2 (Radiation Oncologist) | 2 | DL | 3 | GT |
| 3 (Radiation Oncologist) | 3 | GT | 3 | DL |

*Case 2: Segmentation Failure #1*

In this case, we further investigate an instance where segmentation performance was markedly worse than other model predictions (“Low Performance” example in main manuscript). The patient was a 59-year-old Caucasian male with a right-sided tonsillar HPV positive tumor, stage IVA (T4, N2c, M1), treated with induction chemotherapy (carboplatin+paclitaxel) followed by concurrent chemoradiotherapy (cetuximab-based chemo with volumetric modulated arc radiotherapy). As highlighted in the main manuscript, this case was chosen due to its relatively poor DSC performance overall. Specifically, mean DSC was notably lower for this case when compared to the mean DSC across all cases for the T2 baseline model (0.37 vs. 0.72), T2+T1 model (0.61 vs. 0.73), and ALL model (0.57 vs. 0.71).

While the primary tumor for this case was identified and treated as an OPC, this case is unique among the rest of OPC tumors used to train the models in that the primary tumor spread to areas of the oral cavity and tongue. Since these areas of tumor spread were almost completely unseen by the model in the training process, it can be inferred that model performance would generally be suboptimal. Specifically, it seems this case caused large issues in the T2 model, leading to many spurious voxels in the back of the head to be predicted throughout almost the entire field of view (**Fig. B4**). Importantly these errors were avoided with the addition of supplementary channels in the model input. It is possible the relatively unknown nature of the ground truth segmentation (abnormally large tumor entering oral cavity) caused problems in model optimization for the ground truth T2 baseline model leading to the erroneous model fitting of local minima, which were circumvented through the addition of additional channels. Of note, post-processing techniques such as suppression of all but the largest connected component in a segmentation mask [9] could potentially lessen these errors in future model building approaches.

At the superior-most slices, the T2 baseline model and ALL model erroneously detected false positive voxels in different areas (**Fig. B4, B6**, 1st row); specifically, the T2 model erroneously segmented portions of the epiglottis inferior to the tumor while the ALL model erroneously segmented portions of a metastatic cervical lymph node. Misleading areas of T2 hypointensity within the epiglottis may be a reason for the erroneous segmentation from the T2 model, while confounding signal from parametric maps found in the cervical lymph node may have led to the inaccurate segmentation from the ALL model. These erroneous voxels are not detected by the T2+T1 model (**Fig. B5**, 1st row), highlighting the positive effect introduced by the T1 channel to minimize misleading signal from the T2 model. At the inferior-most slices (**Fig. B4, B5, B6**, 4th row), the T2 model missed areas of ground truth primary tumor almost completely, while the T2+T1 and ALL models were able to accurately detect large portions of the ground truth due to non-conservative predictions (though at the cost of false-positive voxels). However, it should be noted these potentially contested areas correspond to an already challenging ground truth segmentation (further discussed in the Turing test section below).


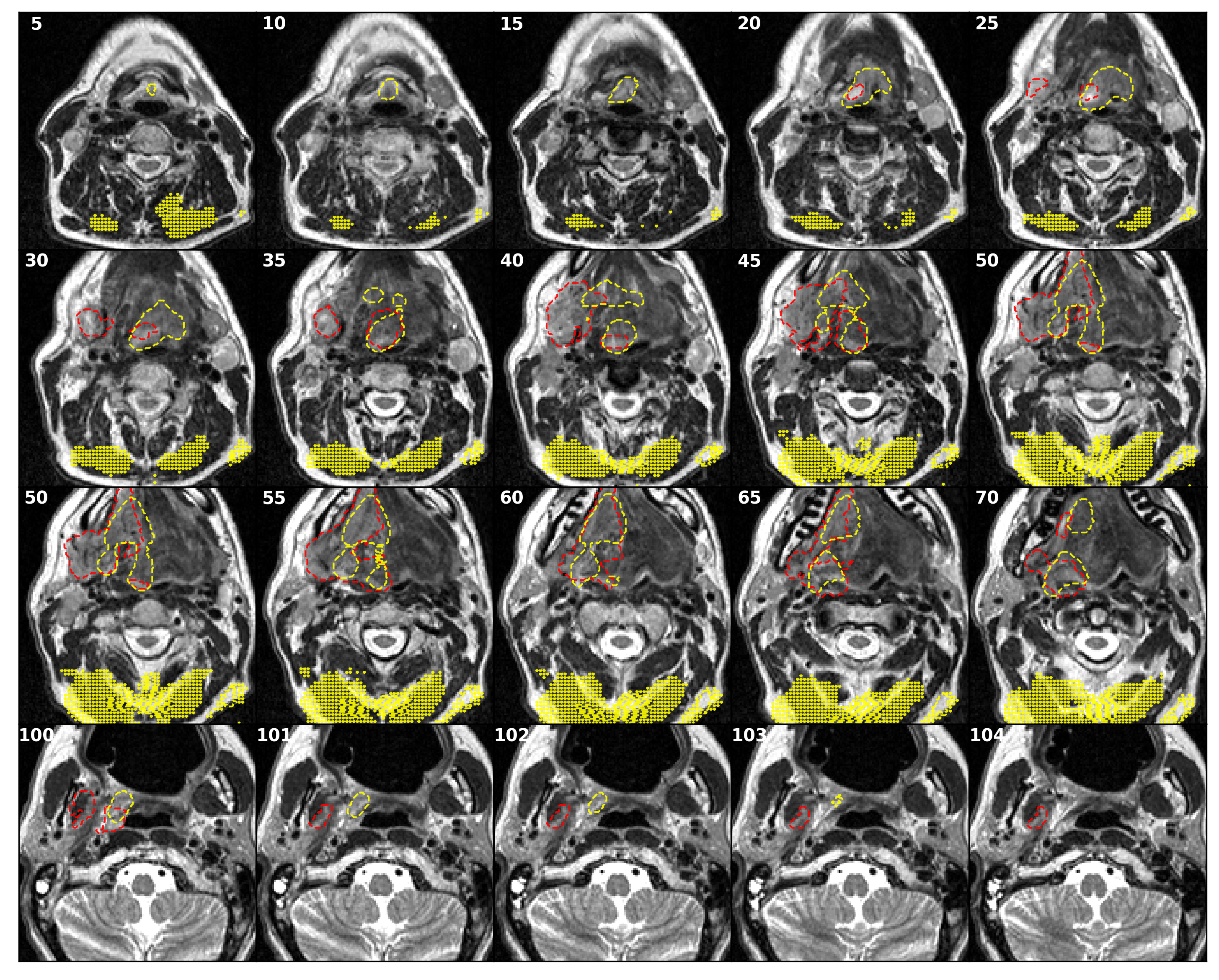


**Figure B4.** Full view 2D axial slice representations of ground truth segmentations (red dotted outline) and predicted segmentations from all 5 channels model (yellow dotted outline) for non-HPV-associated tumor. The slice locations of the segmentations are shown in the top left corners of each image. The DSC for this model was 0.37 for this case.

**
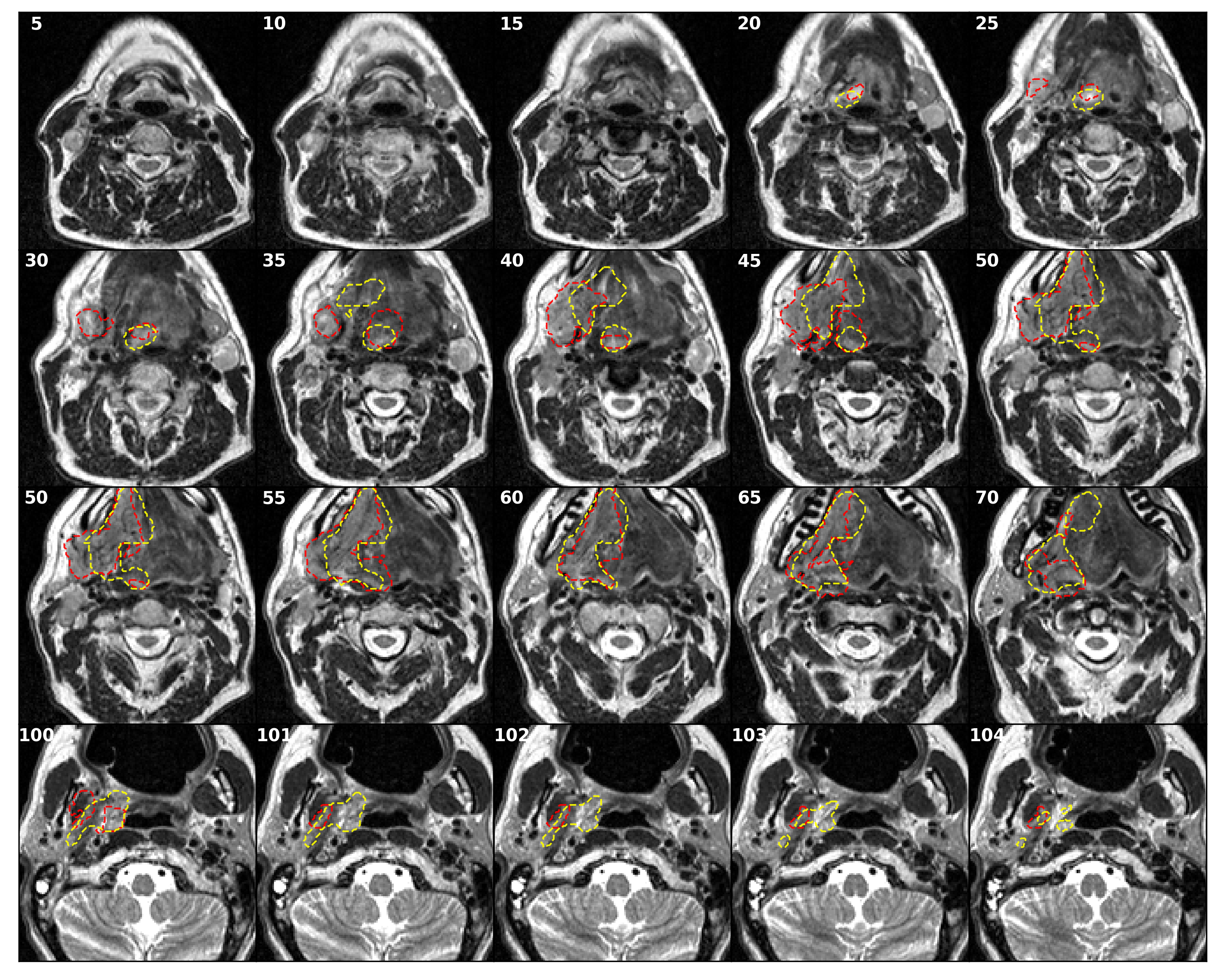
**

**Figure B5.** Full view 2D axial slice representations of ground truth segmentations (red dotted outline) and predicted segmentations from all 5 channels model (yellow dotted outline) for non-HPV-associated tumor. The slice locations of the segmentations are shown in the top left corners of each image. The DSC for this model was 0.61 for this case.


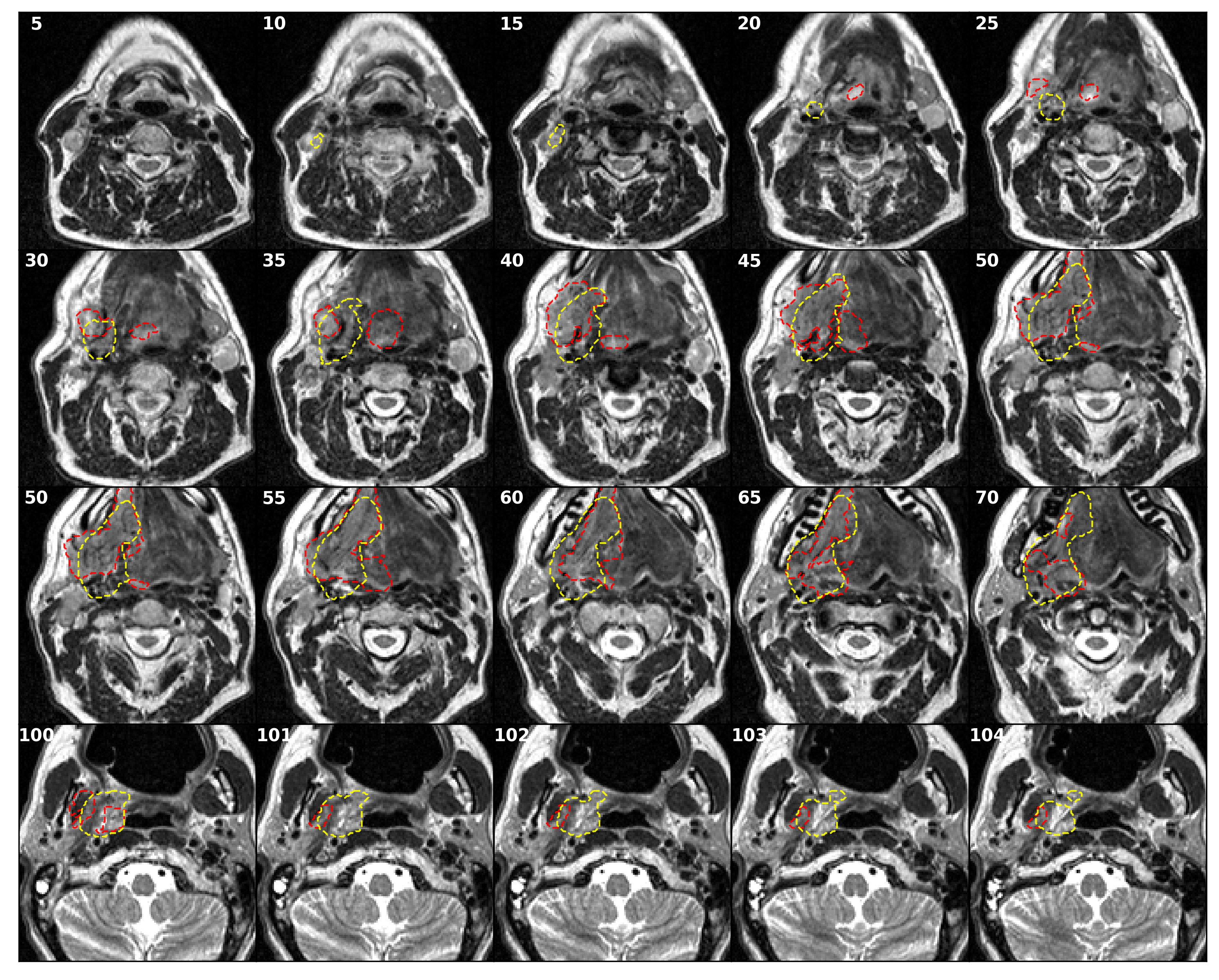


**Figure B6.** Full view 2D axial slice representations of ground truth segmentations (red dotted outline) and predicted segmentations from all 5 channels model (yellow dotted outline) for non-HPV-associated tumor. The slice locations of the segmentations are shown in the top left corners of each image. The DSC for this model was 0.57 for this case.

The clinical evaluation and Turing test results for this case are shown in **Table B2**. Overall, observers gave both the ground truth and model segmentations low scores. Notably, Observer 2 and Observer 3 gave the model segmentations higher scores than the corresponding ground truth. These results point to possibly overall poor segmentation quality of the ground truth, or at the very least an extremely challenging case that warrants a high level of disagreement between observers. Moreover, these results potentially highlight the importance of multi-evaluator segmentation in the generation of ground truth. Future studies should likely consider using consensus methods in the generation of ground truth or algorithmic label fusion approaches (such as Simultaneous Truth and Performance Level Estimation [10]) to avoid the potential for bias (negative or positive) in challenging cases such as these.

**Table B2.** Clinical evaluation and Turing test results for three physician expert observers for segmentation failure case #1. Each observer was asked to score blinded ground truth (GT) or deep learning (DL)-generated segmentations on a 4-point Likert scale (1 = requires corrections, large errors; 2 = requires corrections, minor errors; 3 = clinically acceptable, errors not clinically significant; 4 = clinically acceptable, highly accurate) and asked to identify the source of the segmentation (GT or DL). DL-generated segmentations corresponded to the best DL model tested (T2-weighted + T1-weighted).

| **Observer** | **GT Score** | **GT Source** | **DL Score** | **DL Source** |
| --- | --- | --- | --- | --- |
| 1 (Radiologist) | 1 | DL | 1 | DL |
| 2 (Radiation Oncologist) | 2 | DL | 3 | GT |
| 3 (Radiation Oncologist) | 1 | DL | 2 | DL |

*Case 3: Segmentation Failure #2*

In this case, we investigate an additional instance where segmentation performance was markedly worse than other model predictions. The patient was a 78-year-old Caucasian male with a right-sided tonsillar HPV positive tumor, stage II (T2, N0, M0), treated with concurrent chemoradiotherapy (cetuximab-based chemo with volumetric modulated arc radiotherapy). Of note, this was a recurrent tumor six months post transoral robotic surgery. The DSC performance for this case was relatively poor compared to the average performance for other cases for all models evaluated. Specifically, mean DSC was notably lower for this case when compared to the mean DSC across all cases for the T2 baseline model (0.45 vs. 0.72), T2+T1 model (0.46 vs. 0.73), and ALL model (0.50 vs. 0.71).

At the inferior-most slices, the T2 model and T2+T1 model were completely unable to detect the presence of the tumor for the majority of slices (**Fig. B7, B8**, 1st row). This is odd because the anatomical sequences demonstrated an area of relative hyper and hypointensity (for T2 and T1, respectively) indicative of tumor, but the models were still unable to detect any tumor voxels. It is possible the post-op surrounding scar tissue confounded the ability of these models to discriminate the tumor based on the presented images. Oppositely, the ALL model was able to detect portions of the tumor in the bottom-most slices, but also erroneously detected large portions of the healthy surrounding tissue that was likely post-op scar tissue (**Fig. B9**, 1st row). These areas likely had greater signal on parametric maps, leading to their detection with the ALL model. At slices 40-52 (**Fig. B7, B8, B9**, 2nd row), the three models were able to detect portions of the tumor, but generally under-segmented the tumor. Slightly superiorly (**Fig. B7, B8, B9**, 3rd row) the 3 models started to generate less conservative estimates of tumor voxels, which led to a greater amount of overlap with ground truth at the cost of false-positive predictions; this was most apparent for the ALL model which took the least conservative approach among all methods. At the superior-most slices, the T2 model predictions offer the most reasonable estimates of ground truth (**Fig. B7,** 4th row), while the T2+T1 and ALL models overestimated the degree of tumor spread superiorly (**Fig. B8, B9**, 4th row); the ALL model offered the least conservative segmentations. This may indicate additional channels at superior slices started to overestimate tumor signal. Moreover, as opposed to the other previously described cases, the addition of a greater number of channels did not increase performance for this case. This may be due to this patient generally being an outlier in model training due to their post-op status.


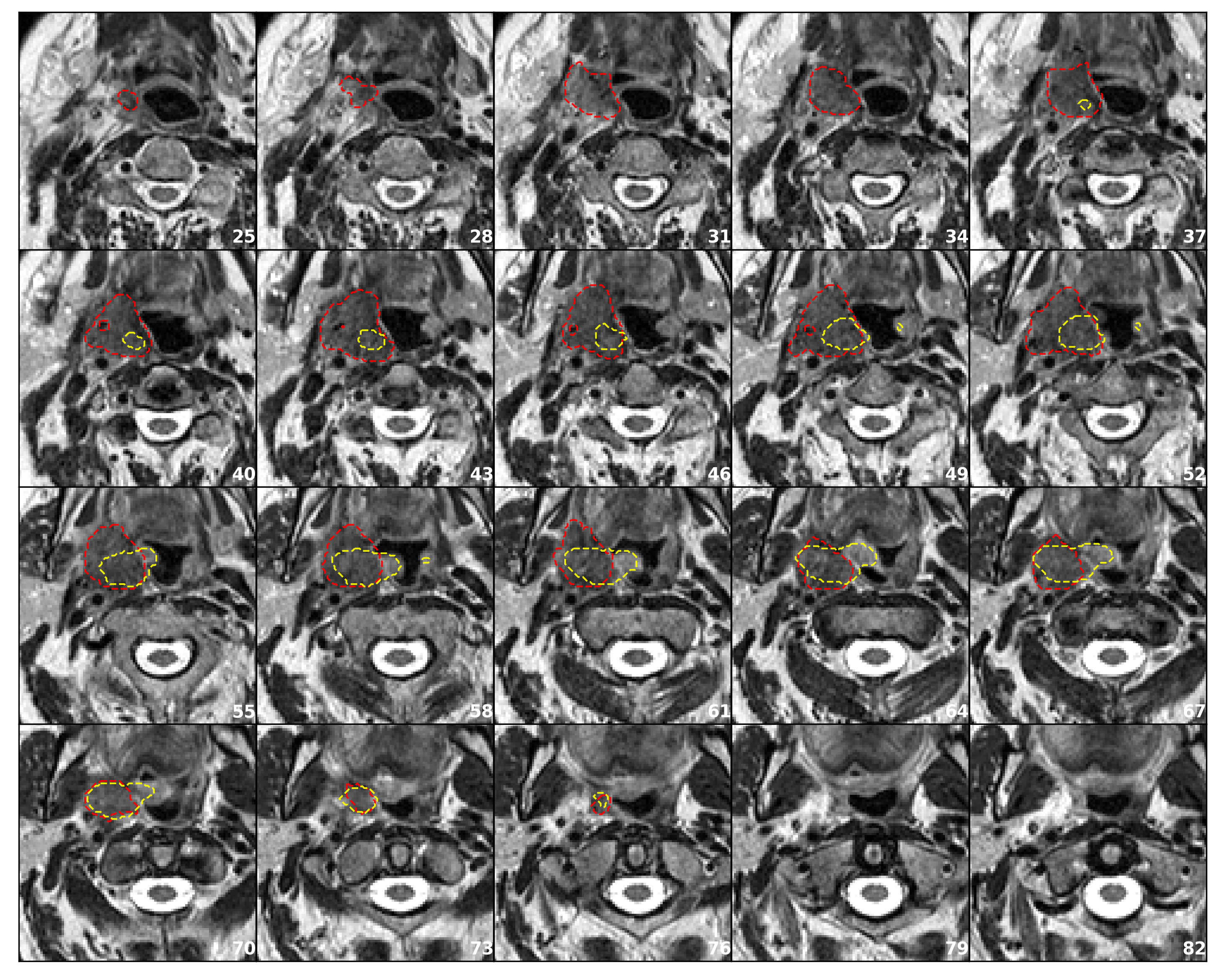


**Figure B7.** Full view 2D axial slice representations of ground truth segmentations (red dotted outline) and predicted segmentations from all 5 channels model (yellow dotted outline) for non-HPV-associated tumor. The slice locations of the segmentations are shown in the bottom right corners of each image. The DSC for this model was 0.45 for this case.


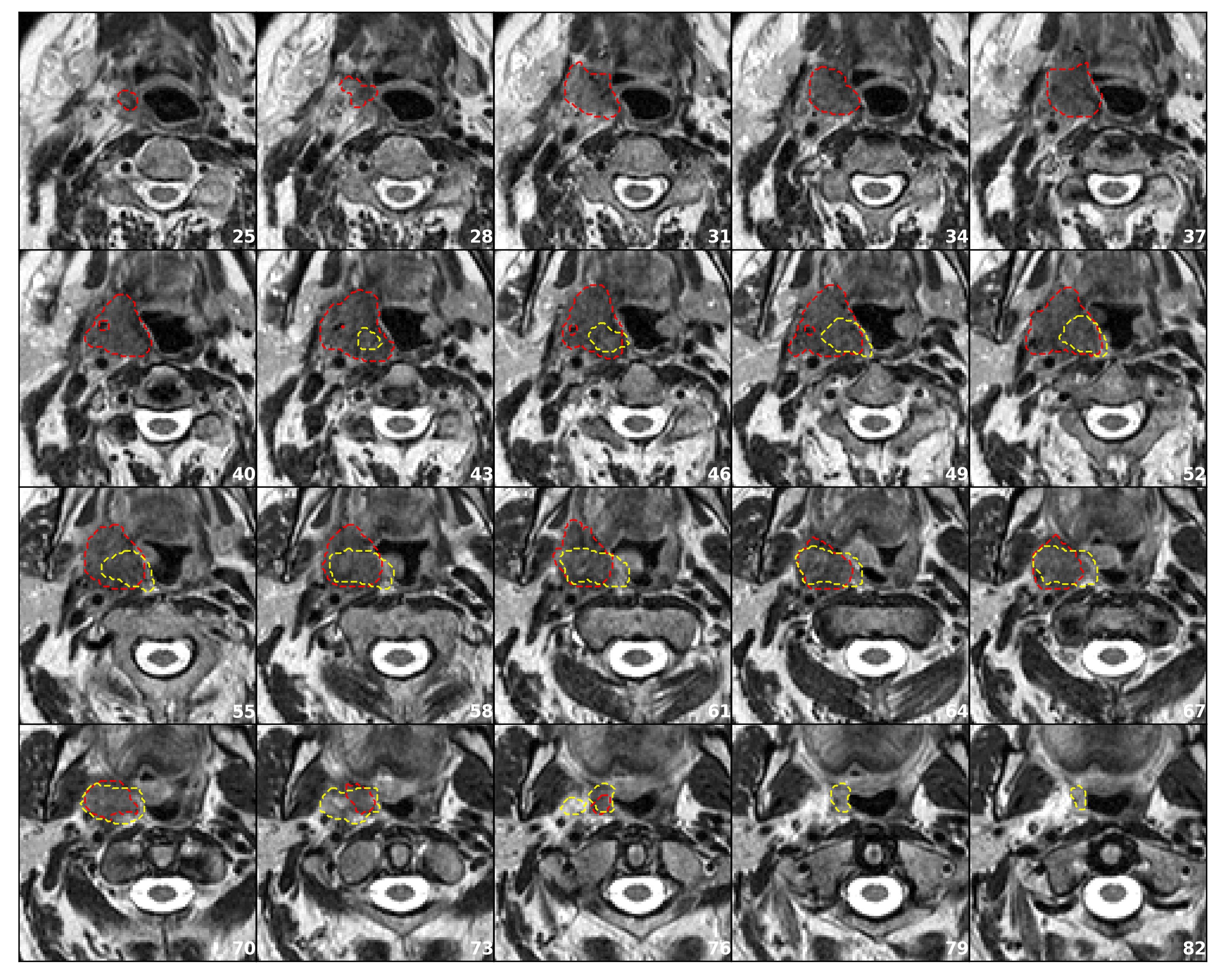


**Figure B8.** Full view 2D axial slice representations of ground truth segmentations (red dotted outline) and predicted segmentations from all 5 channels model (yellow dotted outline) for non-HPV-associated tumor. The slice locations of the segmentations are shown in the bottom right corners of each image. The DSC for this model was 0.46 for this case.


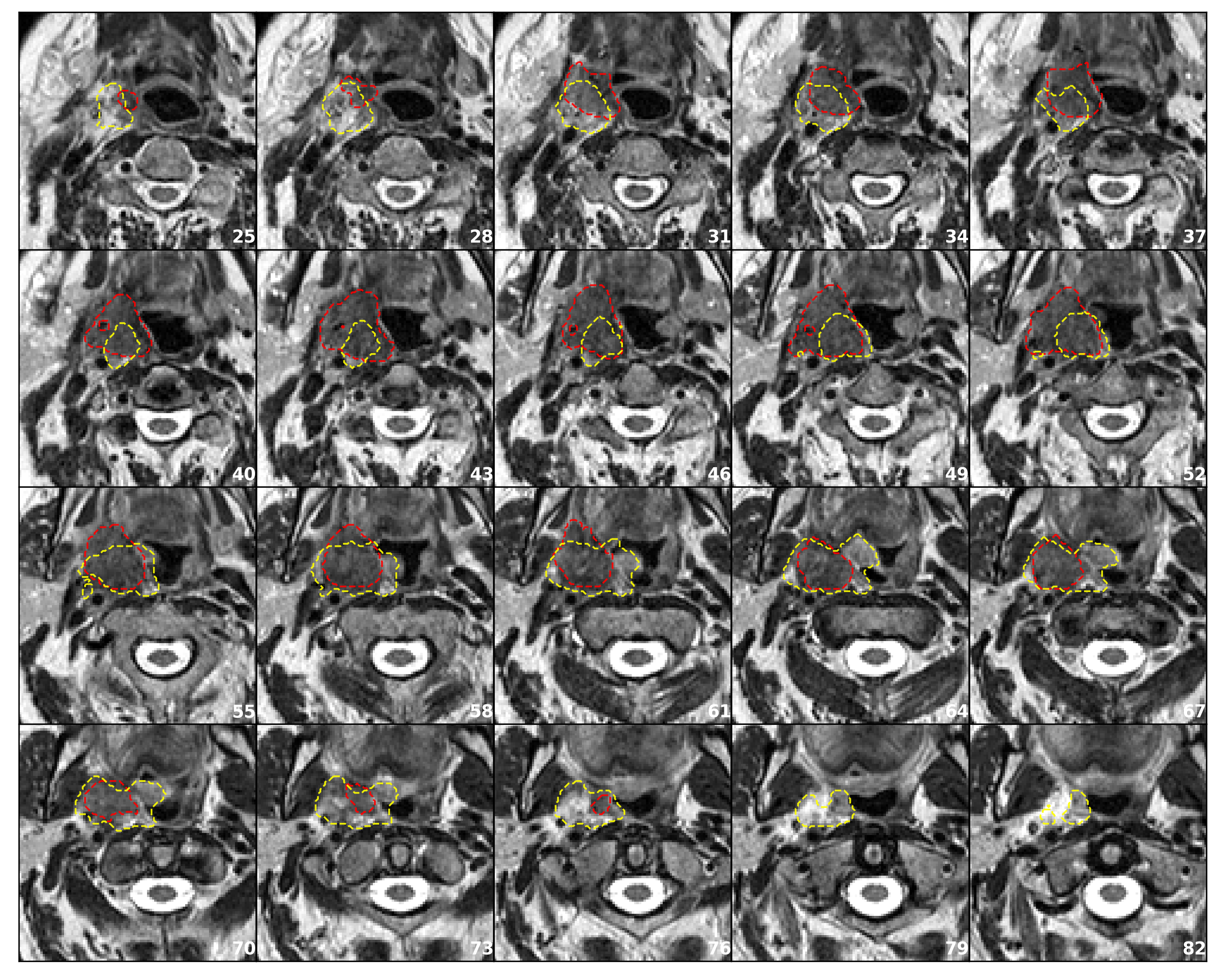


**Figure B9.** Full view 2D axial slice representations of ground truth segmentations (red dotted outline) and predicted segmentations from all 5 channels model (yellow dotted outline) for non-HPV-associated tumor. The slice locations of the segmentations are shown in the bottom right corners of each image. The DSC for this model was 0.50 for this case.

The clinical evaluation and Turing test results for this case are shown in **Table B3**. Overall, observers gave the ground truth segmentation modest scores while the deep learning generated segmentation was graded equivalent or worse than the ground truth. These results are consistent with the generally poor quality of deep learning segmentation based on DSC for this case. Interestingly, all observers thought a human did not generate the segmentations for both ground truth and deep learning. However, unlike failure case #1, the quality of the ground truth segmentation for this case was not as heavily contested, so it can be assumed that factors other than the ground truth segmentation quality (as described above) were the driving factors in poor performance of models.

**Table B3.** Clinical evaluation and Turing test results for three physician expert observers for segmentation failure case #2. Each observer was asked to score blinded ground truth (GT) or deep learning (DL)-generated segmentations on a 4-point Likert scale (1 = requires corrections, large errors; 2 = requires corrections, minor errors; 3 = clinically acceptable, errors not clinically significant; 4 = clinically acceptable, highly accurate) and asked to identify the source of the segmentation (GT or DL). DL-generated segmentations corresponded to the best DL model tested (T2-weighted + T1-weighted).

| **Observer** | **GT Score** | **GT Source** | **DL Score** | **DL Source** |
| --- | --- | --- | --- | --- |
| 1 (Radiologist) | 2 | DL | 1 | DL |
| 2 (Radiation Oncologist) | 2 | DL | 2 | DL |
| 3 (Radiation Oncologist) | 4 | DL | 3 | DL |

**References**

[1] He R, Ding Y, Mohamed ASR, Ng SP, Ger RB, Elhalawani H, et al. Simultaneously spatial and temporal higher-order total variations for noise suppression and motion reduction in DCE and IVIM. Medical Imaging 2020: Image Processing, vol. 11313, International Society for Optics and Photonics; 2020, p. 113132K.

[2] Mongan J, Moy L, Kahn CE. Checklist for Artificial Intelligence in Medical Imaging (CLAIM): A Guide for Authors and Reviewers. Radiology: Artificial Intelligence 2020;2:e200029.

[3] Anderson BM, Wahid KA, Brock KK. Simple Python Module for Conversions Between DICOM Images and Radiation Therapy Structures, Masks, and Prediction Arrays. Pract Radiat Oncol 2021;11:226–9.

[4] Ronneberger O, Fischer P, Brox T. U-Net: Convolutional Networks for Biomedical Image Segmentation. Medical Image Computing and Computer-Assisted Intervention – MICCAI 2015, Springer International Publishing; 2015, p. 234–41.

[5] Naser MA, Deen MJ. Brain tumor segmentation and grading of lower-grade glioma using deep learning in MRI images. Comput Biol Med 2020;121:103758.

[6] Ma N, Li W, Brown R, Wang Y, Gorman B, Behrooz, et al. Project-MONAI/MONAI: 0.5.0. 2021. https://doi.org/10.5281/zenodo.4679866.

[7] He K, Zhang X, Ren S, Sun J. Delving deep into rectifiers: Surpassing human-level performance on imagenet classification. Proceedings of the IEEE international conference on computer vision, 2015, p. 1026–34.

[8] Chan MW, Yu E, Bartlett E, O’Sullivan B, Su J, Waldron J, et al. Morphologic and topographic radiologic features of human papillomavirus-related and -unrelated oropharyngeal carcinoma. Head Neck 2017;39:1524–34.

[9] Ibragimov B, Xing L. Segmentation of organs-at-risks in head and neck CT images using convolutional neural networks. Med Phys 2017;44:547–57.

[10] Warfield SK, Zou KH, Wells WM. Simultaneous truth and performance level estimation (STAPLE): an algorithm for the validation of image segmentation. IEEE Trans Med Imaging 2004;23:903–21.

**Appendix C. Additional Supplementary Figures and Tables**


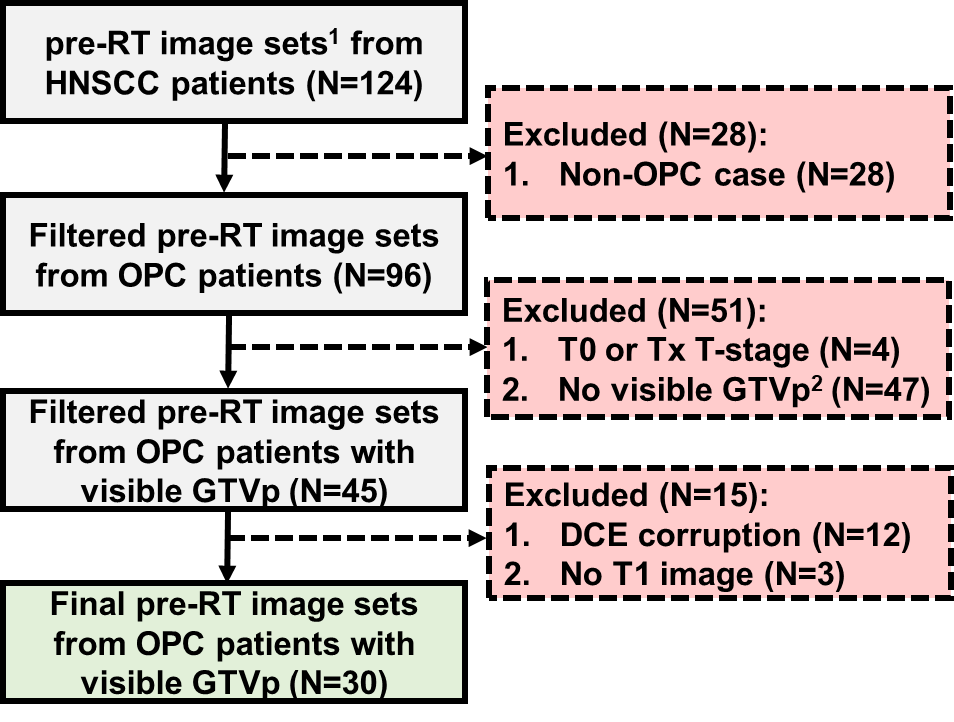


**Figure C1.** Patient selection flow diagram. ^1^ Images corresponded to T2-weighted, T1-weighted, dynamic contrasted enhanced, and diffusion weighted MRI sequences. ^2^ Tumors were not visible due to either surgery or induction chemotherapy before administration of radiotherapy. RT=radiotherapy, HNSCC=head and neck squamous cell carcinoma, OPC=oropharyngeal cancer, GTVp=primary gross tumor volume, DCE=dynamic contrast-enhanced MRI, T1=T1-weighted MRI, mpMRI=multiparametric MRI.


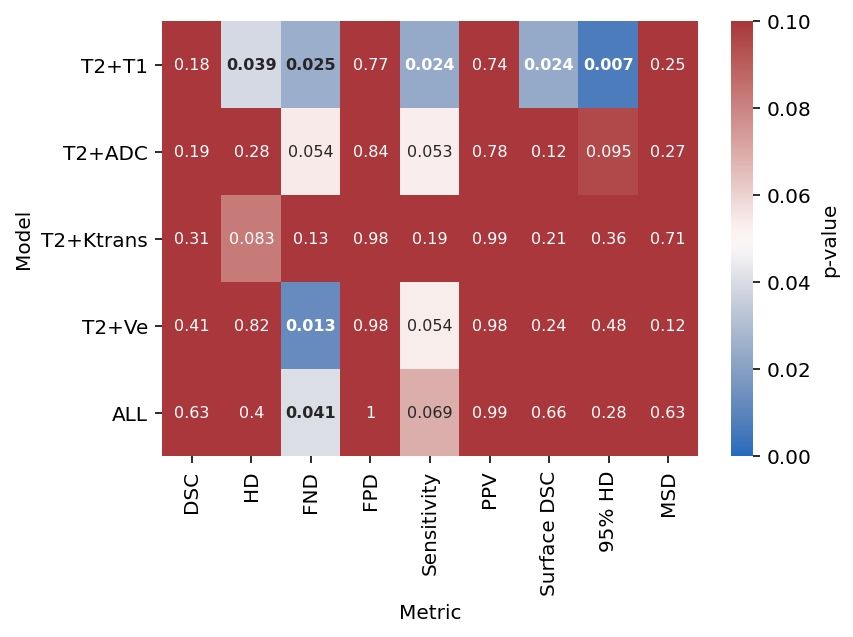


**Figure C2.** Heatmap of Wilcoxon signed-rank tests comparing additional input channel models with the baseline T2 model. Dice similarity coefficient (DSC), sensitivity, positive predictive value (PPV), and surface DSC comparison tests were one-way greater than, while Hausdorff distance (HD), false-negative DSC (FND), false-positive DSC (FPV), 95% HD, and mean surface distance (MSD) were one-way less than. Red corresponds to non-significant p-values, while blue corresponds to significant p-values. T2=T2-weighted MRI, T1=T1-weighted MRI, ADC=apparent diffusion coefficient, Ktrans=volume transfer constant, Ve=extravascular extracellular volume fraction, ALL=all five input channels.

**Table C1.** Patient demographic characteristics. Unless otherwise indicated, data shown correspond to patient number counts. HPV=human papillomavirus, AJCC=American Joint Committee on Cancer. HPV status was determined using in-situ hybridization and p16 immunohistochemistry.

| **Characteristic** | **Value** |
| --- | --- |
| Age (median, range) | 65 (54-78) |
| Sex |  |
| Male | 30 |
| Female | 0 |
| Race |  |
| American Indian | 1 |
| Black/African American | 1 |
| White/Caucasian | 28 |
| Tumor subsite |  |
| Base of tongue | 10 |
| Tonsil | 20 |
| Tumor laterality |  |
| Right | 14 |
| Left | 12 |
| Bilateral | 3 |
| Midline | 1 |
| HPV status |  |
| Negative | 1 |
| Positive | 29 |
| T-category |  |
| T1 | 6 |
| T2 | 14 |
| T3 | 3 |
| T4 | 7 |
| N-category |  |
| N0 | 1 |
| N1 | 18 |
| N2a | 2 |
| N2b | 4 |
| N2c | 4 |
| N3 | 1 |
| AJCC stage |  |
| I | 10 |
| II | 5 |
| III | 6 |
| IVA | 8 |
| IVC | 1 |

**Table C2.** MRI sequence acquisition parameters. T2=T2-weighted MRI, T1=T1-weighted MRI, DCE=dynamic contrast enhanced MRI, DWI=diffusion weighted imaging MRI.

| **Acquisition Parameter** | **T2** | **T1** | **DCE** | **DWI** |
| --- | --- | --- | --- | --- |
| Repetition time (ms) | 4800.00 | 7.11 | 8.60 | 5400.00 |
| Echo time (ms) | 80.00 | 2.39 | 1.90 | 50.00 |
| Echo train length | 15 | 2 | 1 | 15 |
| Flip angle (°) | 180 | 10 | 15 | 120 |
| Slice thickness (mm) | 2.00 | 1.00 | 1.00 | 4.00 |
| In-plane resolution (mm) | 0.50 | 1.00 | 1.00 | 2.00 |
| Acquisition matrix | 256x230 | 256x256 | 128x83 | 128x128 |
| Pixel bandwidth (Hz/px) | 300.00 | 405.00 | 600.00 | 1220.00 |
| Number of averages | 1 | 2 | 1 | 8 |
| b-values (s/mm^2) | NA | NA | NA | 0, 800 |

**Table C3.** Mann-Whitney U tests between performance on base of tongue and tonsil subsites for each model for various evaluation metrics. Evaluation metrics correspond to Dice similarity coefficient (DSC), Hausdorff distance (HD), false-negative DSC (FND), false-positive DSC (FPD), sensitivity, positive predictive value (PPV), surface DSC, 95% HD, and mean surface distance (MSD). All p-values were > 0.05. T2=T2-weighted MRI, T1=T1-weighted MRI, ADC=apparent diffusion coefficient, Ktrans=volume transfer constant, Ve=extravascular extracellular volume fraction, ALL=all five input channels.

| **Metric** | **Model** | **p-value** |
| --- | --- | --- |
| DSC | T2 | 0.404 |
| HD | T2 | 0.208 |
| FND | T2 | 0.404 |
| FPD | T2 | 0.214 |
| Sensitivity | T2 | 0.456 |
| PPV | T2 | 0.262 |
| Surface DSC | T2 | 0.404 |
| 95% HD | T2 | 0.346 |
| MSD | T2 | 0.456 |
| DSC | T2+T1 | 0.491 |
| HD | T2+T1 | 0.387 |
| FND | T2+T1 | 0.184 |
| FPD | T2+T1 | 0.371 |
| Sensitivity | T2+T1 | 0.291 |
| PPV | T2+T1 | 0.346 |
| Surface DSC | T2+T1 | 0.208 |
| 95% HD | T2+T1 | 0.387 |
| MSD | T2+T1 | 0.422 |
| DSC | T2+ADC | 0.491 |
| HD | T2+ADC | 0.354 |
| FND | T2+ADC | 0.284 |
| FPD | T2+ADC | 0.184 |
| Sensitivity | T2+ADC | 0.371 |
| PPV | T2+ADC | 0.241 |
| Surface DSC | T2+ADC | 0.172 |
| 95% HD | T2+ADC | 0.202 |
| MSD | T2+ADC | 0.291 |
| DSC | T2+Ktrans | 0.491 |
| HD | T2+Ktrans | 0.430 |
| FND | T2+Ktrans | 0.338 |
| FPD | T2+Ktrans | 0.189 |
| Sensitivity | T2+Ktrans | 0.354 |
| PPV | T2+Ktrans | 0.422 |
| Surface DSC | T2+Ktrans | 0.208 |
| 95% HD | T2+Ktrans | 0.131 |
| MSD | T2+Ktrans | 0.422 |
| DSC | T2+Ve | 0.474 |
| HD | T2+Ve | 0.322 |
| FND | T2+Ve | 0.330 |
| FPD | T2+Ve | 0.338 |
| Sensitivity | T2+Ve | 0.422 |
| PPV | T2+Ve | 0.371 |
| Surface DSC | T2+Ve | 0.387 |
| 95% HD | T2+Ve | 0.322 |
| MSD | T2+Ve | 0.474 |
| DSC | ALL | 0.404 |
| HD | ALL | 0.122 |
| FND | ALL | 0.322 |
| FPD | ALL | 0.255 |
| Sensitivity | ALL | 0.422 |
| PPV | ALL | 0.262 |
| Surface DSC | ALL | 0.422 |
| 95% HD | ALL | 0.430 |
| MSD | ALL | 0.354 |
